# Microsatellites reveal a strong subdivision of genetic structure in Chinese populations of the mite Tetranychus urticae Koch (Acari: Tetranychidae)

**DOI:** 10.1186/1471-2156-13-8

**Published:** 2012-02-21

**Authors:** Jing-Tao Sun, Chunlan Lian, Maria Navajas, Xiao-Yue Hong

**Affiliations:** 1 Department of Entomology, Nanjing Agricultural University, Nanjing, Jiangsu 210095, China; 2 Asian Natural Environmental Science Center, The University of Tokyo, Midori-cho 1-1-8, Nishitokyo-shi, Tokyo 188-0002, Japan; 3 INRA, CBGP, Campus International de Baillarguet, CS 30016, 34988 Montferrier-sur-Lez, France

**Keywords:** Two-spotted spider mite, Green form, Red form, Genetic diversity, Null alleles

## Abstract

**Background:**

Two colour forms of the two-spotted spider mite (*Tetranychus urticae *Koch) coexist in China: a red (carmine) form, which is considered to be native and a green form which is considered to be invasive. The population genetic diversity and population genetic structure of this organism were unclear in China, and there is a controversy over whether they constitute distinct species. To address these issues, we genotyped a total of 1,055 individuals from 18 red populations and 7 green populations in China using eight microsatellite loci.

**Results:**

We identified 109 alleles. We found a highly significant genetic differentiation among the 25 populations (global *F*_ST _= 0.506, global *F*_ST _^{*ENA*} ^= 0.473) and a low genetic diversity in each population. In addition, genetic diversity of the red form mites was found to be higher than the green form. Pearson correlations between statistics of variation (*AR *and *H*_E_) and geographic coordinates (latitude and longitude) showed that the genetic diversity of the red form was correlated with latitude. Using Bayesian clustering, we divided the Chinese mite populations into five clades which were well congruent with their geographic distributions.

**Conclusions:**

Spider mites possess low levels of genetic diversity, limit gene flow between populations and significant and IBD (isolation by distance) effect. These factors in turn contribute to the strong subdivision of genetic structure. In addition, population genetic structure results don't support the separation of the two forms of spider mite into two species. The morphological differences between the two forms of mites may be a result of epigenetic effects.

## Background

The phytophagous two-spotted spider mite *Tetranychus urticae *Koch is a serious pest of various agricultural plants including fruit trees, vegetables, ornamentals and agricultural crops. Owing to its rapid development and high reproductive capacities, and ability to feed on more than 900 plants [1], *T*. *urticae *has spread worldwide. Despite the importance of this pest, the population genetic structure of this mite has been unclear in China. A better understanding of population genetic structure could help to manage mite populations by providing more reliable estimates of population dynamics and the risk of spreading acaricide resistance genes. The population genetic structure of an organism is determined by various factors, such as geographical barriers, ecological difference, and historical processes, as well as the dispersal ability of the species. *T*. *urticae *has multiple dispersal mechanisms. Spider mites are wingless and usually rely on crawling for their dispersal [2]. But they can also be carried for long distance by the wind and by human activities [3,4]. Due to the complex dispersal mechanisms of *T*. *urticae*, the population structure and diversity would be complex. Two studies have used molecular markers to investigate the genetic structure of *T. urticae *in China [5,6]. However, due to the limited number of markers used to analyse geographic populations and individuals, the information that they provided was insufficient to understand the genetic diversity and population structure clearly. To obtain further insights into the population genetic structure of *T*. *urticae*, a more systematic and wide scope study based on more microsatellite markers using more powerful Statistical analysis methods are needed.

In addition to questions about the population structure and genetic diversity of *T. urticae *in China, there is a big question about its taxonomy. *T. urticae *has two forms: a green form that occurs in temperate and cold areas, and a red (carmine) non-diapausing form (also called *T*. *cinnabarinus *(Boisduval)) that occurs in warmer areas [7]. Although distinguishable by body colour and the shape of the male aedeagus, the two forms are polymorphic and have significant intra-specific variation among populations by virtue of their different host plants and different habitats. Furthermore, there is a partial to complete reproductive incompatibility between them [8,9]. Therefore, whether they constitute one or two species has been debated for over 20 years. In China, the two forms coexist. However, the red form is considered to be native, while the green form, which was first reported in 1983 in Beijing, is considered to be invasive [10]. The carmine form is distributed throughout China. The green form has recently expanded its distribution from its putative source area in Beijing to many parts of the country, including Hebei, Shanghai, Liaoning, Jilin, Gansu, Ningxia, Henan, Anhui and Jiangsu provinces and elsewhere [10,11]. Therefore, information with respect to the genetic relationship of the two forms of *T*. *urticae *would help to clarify the longstanding issue of their species status.

In this study, two new microsatellite markers developed by ourselves together with six previously defined loci [12,13] were used to gain further insights into the genetic structure of *T. urticae *in China. Our main goals were to unveil the genetic structure across the known range of *T. urticae *in China, and clarify the taxonomic status of red and green spider mites from a population genetic structure perspective.

## Results

### Microsatellite development

Of the 180 clones sequenced, 127 were discarded because the repetition pattern was too short. Of the 53 remaining microsatellites, 44 appeared to be either monomorphic or to have an unclear banding pattern in gels, and seven would not amplify in each of the 40 adult females. Only two microsatellites isolated here (clones TECI104 and TECI08, GenBank accession numbers GU068508 and GU068509, respectively) revealed polymorphism and were used.

### Genetic variation

The allelic richness based on a minimum population size of 34 diploid individuals ranged from 2.14 to 6.23 with an average value of 3.89 for every population (Table 1). In general, the observed and expected heterozygosity values were very low, ranging from 0.111 to 0.454 and 0.181-0.754 respectively (Table 1). Tests for linkage disequilibrium between all pairs of loci across populations using FSTAT found no significant genotypic disequilibrium in the pooled data (*P *> 0.05 for all after Bonferroni corrections). MICRO-CHECKER software identified the presence of null alleles. The frequencies of null alleles per locus per population ranged from 0 to 0.434 (only four cases > 0.4). In 179 of 200 locus-population combinations, the frequencies of null alleles were lower than 0.200. There was a strong overall heterozygote deficit, with *f *= 0.272 (95% confidence interval, 0.115-0.391). Nineteen of 25 populations showed a significant deficit of heterozygotes (Table 1). Most populations and most loci did not meet the criteria for Hardy-Weinberg equilibrium. Only two green-form spider mite populations, 5 (G) and 4 (G), were in Hardy-Weinberg equilibrium. After correcting the data set for null alleles using the EM algorithm, both the observed and expected heterozygosity values were higher than the raw data ranging from 0.239 to 0.815 and 0.223-0.789 respectively (Table 1). No significant heterozygote deficiency was detected (*f *= - 0.049, 95% confidence interval, -0.058-0.006). Of 200 locus-population combinations, 192 were in Hardy-Weinberg equilibrium. Only three populations, 20 (R), 1 (G) and 4 (G), were not in Hardy-Weinberg due to heterozygote excess at 2-3 loci.

**Table 1:** Populations and genetic diversity measures estimated using eight microsatellites in each of 25 *Tetranychus urticae *populations

POPS	POP Code	Host plant	n	*A*	*AR*	^R^*H*_O_/^C^*H*_O_	^R^*H*_E_/^C^*H*_E_	^R^*F*_IS/_/^C^*F*_IS_
JGDN(R)	1 (R)	Eggplant	41	3.63	2.79	0.204/0.274	0.255/0.263	0.211*/-0.045

HLJYC(R)	2 (R)	Cowpea	44	4.00	3.21	0.119/0.347	0.235/0.341	0.501*/-0.014

LNSY(R)	3 (R)	Cowpea	39	4.63	4.08	0.240/0.410	0.354/0.432	0.332*/0.061

SDJN(R)	6 (R)	Cotton	44	4.63	3.65	0.409/0.455	0.383/0.413	-0.056/-0.069

HBQX(R)	7 (R)	Cotton	42	5.13	3.44	0.408/0.528	0.451/0.510	0.107*/-0.024

HNZZ(R)	8 (R)	Cotton	42	6.75	5.27	0.390/0.537	0.493/0.527	0.221*/-0.008

SXYC(R)	9 (R)	Cotton	42	3.88	3.27	0.259/0.393	0.311/0.389	0.179*/0.018

SXYL(R)	10 (R)	Apple	40	5.75	5.38	0.278/0.709	0.600/0.694	0.545*/-0.021

GSTS(R)	11 (R)	Bean	41	4.88	4.60	0.210/0.530	0.409/0.539	0.495*/0.020

SHNH(R)	12 (R)	Cotton	43	3.63	3.46	0.404/0.482	0.445/0.487	0.104*/0.003

JSZJ(R)	13 (R)	Bean	44	6.63	5.92	0.415/0.681	0.672/0.713	0.391*/0.047

ZJCX(R)	14 (R)	Cotton	44	5.34	4.46	0.306/0.519	0.430/0.514	0.298*/-0.006

AHAQ(R)	15 (R)	Cotton	44	6.50	5.81	0.381/0.727	0.632/0.686	0.407*/-0.061

JXJJ(R)	16 (R)	Cotton	42	4.25	3.22	0.226/0.277	0.263/0.269	0.151*/-0.035

HBWH(R)	17 (R)	Cotton	42	7.12	6.23	0.340/0.815	0.754/0.789	0.557*/-0.034

HNCS(R)	18 (R)	Cowpea	45	3.75	3.51	0.144/0.481	0.327/0.483	0.566*/0.006

SCMS(R)	19 (R)	Cotton	41	4.75	4.22	0.440/0.554	0.503/0.527	0.139*/-0.048

YNYL(R)	20 (R)	Bean	41	4.5	3.53	0.454/0.540	0.435/0.446	-0.032/-0.187*

JGDN(G)	1 (G)	Eggplant	44	3.00	2.96	0.412/0.500	0.431/0.456	0.057/-0.121*

LNXC(G)	4 (G)	Apple	36	3.88	2.97	0.356/0.430	0.362/0.379	0.031/-0.139*

HBCL(G)	5 (G)	Apple	44	2.75	2.14	0.222/0.239	0.219/0.223	-0.001/-0.056

SDJN(G)	6 (G)	Apple	39	4.63	4.36	0.292/0.458	0.389/0.455	0.262*/0.041

HNZZ(G)	8 (G)	Apple	44	2.75	2.65	0.111/0.287	0.181/0.286	0.396*/0.013

GSTS(G)	11 (G)	Apple	43	3.25	2.92	0.151/0.283	0.209/0.278	0.287*/-0.023

XJZN(G)	21 (G)	Apple	43	3.88	3.12	0.285/0.349	0.288/0.323	0.022/-0.074

* *p *< 0.05. (Apple: *Malus pumila *Mill, Bean: *Phaseolus vulgaris *L., Cotton: *Gossypium spp*., Cowpea: *Vignaunguiculata *(L.) Walp, Eggplant: *Solanum melongena *L.)

Sample size (*n*), number of alleles per locus (*A*), allelic Richness for standardized samples of 34 individuals (*AR*), observed heterozygosity calculated by the raw data (^R^*H*_O_), observed heterozygosity calculated by the corrected data (^C^*H*_O_), expected heterozygosity calculated by the raw data (^R^*H*_E_), corrected expected heterozygosity (^C^*H*_E_), fixation index calculated by the raw data (^R^*F*_IS_), fixation index calculated by the corrected data (^C^*F*_IS_)

**Figure 1:**
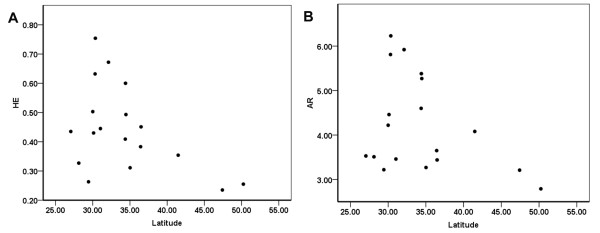
**Pearson correlations between statistics of variation (**^**R**^***H***_**E **_**and *AR*) and geographic latitude**. (**A**) Expected heterozygosity (^R^*H*_E_); R = -0.476, *P *< 0.05. (**B**) Allele richness (*AR*); R = -0.368, *P *= 0.067.

A Pearson correlation analysis showed that ^R^*H*_E _and ^C^*H*_E _of the red form mite were both significantly and negatively correlated with latitude (for ^R^*H*_E_: R = -0.476, *P *< 0.05, Figure 1A; for ^C^*H*_E_: R = -0.469, *P *< 0.05, Additional file 1). *AR *tended to be higher at lower latitudes, but the correlation was not significant (R = -0.368, *P *= 0.067; Figure 1B). Further stepwise regression analysis of *AR *and *H*_E _showed that *H*_E _significantly contributed to the stepwise regression equation (for ^R^*H*_E_: R^2 ^= 0.227, F = 4.697, *P *< 0.001; for ^C^*H*_E_: R^2 ^= 0.220, F = 4.503, *P *< 0.05) but that *AR *did not contribute significantly (R^2 ^= 0.135, F = 2.505, *P *= 0.133). There was no evidence that either *AR *or *H*_E _of the red form mite was related to longitude. However, for the green form mite, neither latitude nor longitude was correlated with *AR *or *H*_E_. This suggests that the intra-population genetic diversity of the red form mites was negatively correlated with latitude. However, the intra-population genetic diversity of the green form mites was clearly not correlated with latitude.

### Population genetic structure

The overall *F*_ST _value (*F*_ST _= 0.506; 95% confidence interval 0.447-0.564) indicated a high level of population differentiation. Pairwise estimates of *F*_ST _calculated between pairs of populations showed that most tests for population differentiation were significant (Table 2). The only two populations that were not differentiated were 3 (R) and 11 (R) (*F*_ST _= 0.042). Thirty-five pairwise populations showed moderate genetic differentiation (pairwise *F*_ST _< 0.25). The rest of the pairwise populations displayed high genetic differentiation (pairwise *F*_ST _ranged from 0.255 to 0.785). The presence of null alleles caused the level of pairwise population differentiation to be overestimated. But the level of population differentiation was also high with global *F*_ST _^{*ENA*} ^= 0.473 (95% confidence interval 0.418-0.524). In the unbiased *F*_ST _^{*ENA*} ^data set, 42 pairwise populations showed moderate genetic differentiation (pairwise *F*_ST _^{*ENA*} ^< 0.25, Additional file 2). The rest of the pairwise populations displayed high genetic differentiation (pairwise *F*_ST _^{*ENA*} ^ranged from 0.255 to 0.752).

**Table 2:** Pairwise *F*_ST _values between all populations (lower-left matrix) and their significance (upper-right matrix)

population	1(R)	2(R)	3(R)	6(R)	7(R)	8(R)	9(R)	10(R)	11(R)	12(R)	13(R)	14(R)	15(R)	16(R)	17(R)	18(R)	19(R)	20(R)	1(G)	4(G)	5(G)	6(G)	8(G)	11(G)
1(R)		*	*	*	*	*	*	*	*	*	*	*	*	*	*	*	*	*	*	*	*	*	*	*

2(R)	0.707	*	*	*	*	*	*	*	*	*	*	*	*	*	*	*	*	*	*	*	*	*	*	*

3(R)	0.635	0.685	*	*	*	*	*	*	NS	*	*	*	*	*	*	*	*	*	*	*	*	*	*	*

6(R)	0.624	0.681	0.214	*	*	*	*	*	*	*	*	*	*	*	*	*	*	*	*	*	*	*	*	*

7(R)	0.597	0.616	0.159	0.153	*	*	*	*	*	*	*	*	*	*	*	*	*	*	*	*	*	*	*	*

8(R)	0.563	0.646	0.179	0.054	0.146	*	*	*	*	*	*	*	*	*	*	*	*	*	*	*	*	*	*	*

9(R)	0.658	0.717	0.352	0.316	0.378	0.306	*	*	*	*	*	*	*	*	*	*	*	*	*	*	*	*	*	*

10(R)	0.467	0.547	0.121	0.108	0.115	0.071	0.269	*	*	*	*	*	*	*	*	*	*	*	*	*	*	*	*	*

11(R)	0.593	0.649	0.042	0.143	0.111	0.101	0.303	0.101	*	*	*	*	*	*	*	*	*	*	*	*	*	*	*	*

12(R)	0.622	0.649	0.544	0.539	0.505	0.483	0.576	0.359	0.520	*	*	*	*	*	*	*	*	*	*	*	*	*	*	*

13(R)	0.398	0.521	0.210	0.158	0.189	0.142	0.266	0.063	0.188	0.354	*	*	*	*	*	*	*	*	*	*	*	*	*	*

14(R)	0.629	0.630	0.541	0.527	0.497	0.477	0.575	0.356	0.515	0.134	0.350	*	*	*	*	*	*	*	*	*	*	*	*	*

15(R)	0.517	0.532	0.409	0.415	0.361	0.358	0.454	0.247	0.381	0.179	0.235	0.205	*	*	*	*	*	*	*	*	*	*	*	*

16(R)	0.736	0.757	0.649	0.680	0.630	0.628	0.702	0.488	0.622	0.486	0.497	0.566	0.415	*	*	*	*	*	*	*	*	*	*	*

17(R)	0.410	0.365	0.238	0.261	0.210	0.211	0.318	0.097	0.212	0.255	0.118	0.245	0.138	0.314	*	*	*	*	*	*	*	*	*	*

18(R)	0.687	0.688	0.607	0.636	0.587	0.585	0.658	0.443	0.579	0.447	0.461	0.527	0.381	0.076	0.264	*	*	*	*	*	*	*	*	*

19(R)	0.597	0.598	0.439	0.497	0.427	0.447	0.516	0.296	0.414	0.439	0.332	0.468	0.321	0.347	0.186	0.317	*	*	*	*	*	*	*	*

20(R)	0.640	0.646	0.588	0.581	0.551	0.522	0.612	0.387	0.553	0.406	0.403	0.426	0.301	0.412	0.241	0.358	0.292	*	*	*	*	*	*	*

1(G)	0.610	0.627	0.568	0.560	0.534	0.506	0.559	0.381	0.545	0.431	0.387	0.433	0.336	0.520	0.276	0.482	0.412	0.358	*	*	*	*	*	*

4(G)	0.664	0.664	0.621	0.601	0.580	0.559	0.605	0.444	0.592	0.538	0.437	0.544	0.442	0.634	0.346	0.572	0.481	0.513	0.283	*	*	*	*	*

5(G)	0.761	0.749	0.710	0.684	0.665	0.641	0.694	0.530	0.681	0.609	0.532	0.613	0.507	0.705	0.425	0.624	0.571	0.554	0.287	0.417	*	*	*	*

6(G)	0.662	0.628	0.597	0.582	0.547	0.538	0.610	0.433	0.564	0.535	0.429	0.531	0.427	0.626	0.327	0.569	0.459	0.493	0.291	0.283	0.373	*	*	*

8(G)	0.769	0.785	0.715	0.696	0.680	0.657	0.718	0.545	0.685	0.609	0.539	0.625	0.546	0.754	0.482	0.684	0.619	0.618	0.557	0.504	0.666	0.510	*	*

11(G)	0.762	0.749	0.708	0.686	0.663	0.639	0.694	0.533	0.679	0.595	0.532	0.596	0.490	0.727	0.434	0.669	0.590	0.530	0.336	0.464	0.558	0.569	0.747	*

21(G)	0.708	0.738	0.656	0.640	0.628	0.592	0.657	0.461	0.631	0.516	0.473	0.535	0.449	0.633	0.390	0.575	0.512	0.483	0.301	0.405	0.549	0.478	0.623	0.503

* *P *< 0.05 after sequential Bonferroni correction; NS, nonsignificant population differentiation

**Figure 2:**
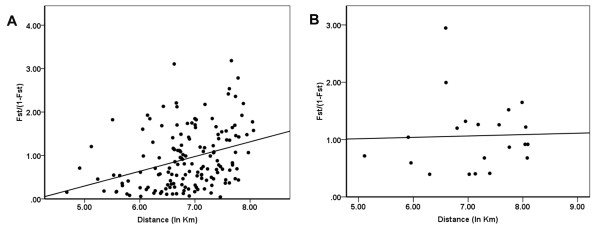
**Scatter plots of genetic distance vs. geographical distance for pairwise population comparisons**. (**A**) Red form mite populations (R^2 ^= 0.1129, *P *= 0.0050; 1000 permutations). (**B**) Green form mite populations (R^2 ^= 0.01, *P *= 0.428; 1000 permutations).

Isolation by distance (IBD) was significant in the populations of the carmine mite [regression of *F*_ST_/(1-*F*_ST_) with ln (distance), Mantel test after 1000 permutations, (18 populations, R^2 ^= 0.1129, *P *= 0.0070; Figure 2A)]. However, the IBD of the populations of the green mite was not significant (7 populations, R^2 ^= 0.001, *P *= 0.42800; Figure 2B). Similar results for *F*_ST _^{*ENA*} ^confirmed that an IBD effect existed in the carmine mite populations (R^2 ^= 0.1143, *P *= 0.018; Additional file 3) and didn't exist in the green mite populations (R^2 ^= 0.0396, *P *= 0.416; Additional file 3).

**Figure 3:**
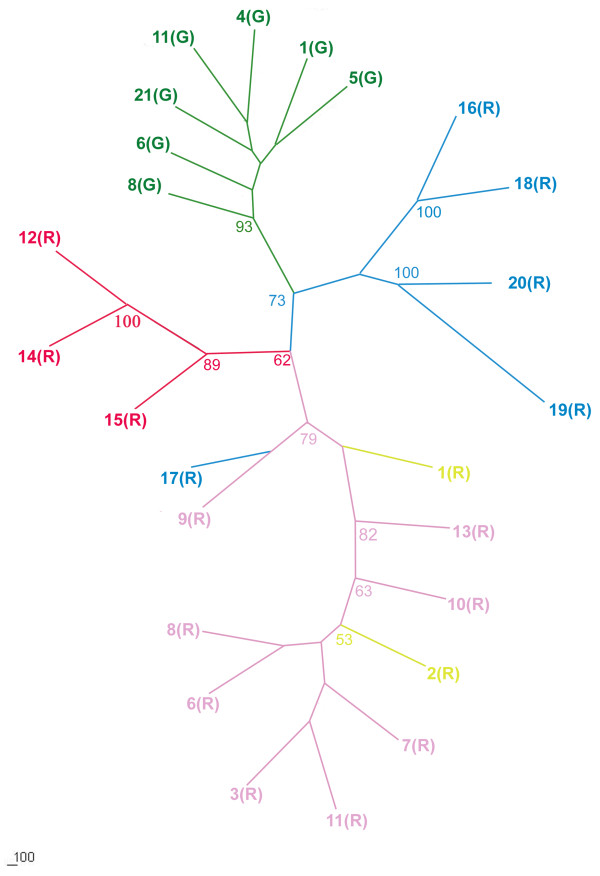
**Consensus neighbour-joining tree based on DCE distances**. Only Bootstrap values >50 are indicated at each node. The colour of clades was selected according to the colour of five clusters inferred by structure when K = 5.

**Figure 4:**
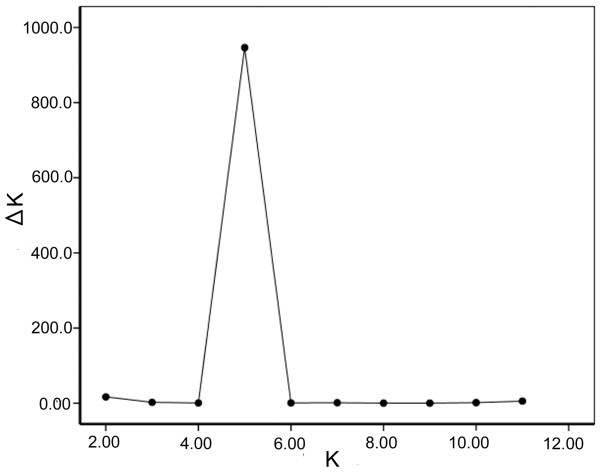
**Scatter plots of ΔK**. ΔK is based on the rate of change of ln *P*(*X*/*K*) between successive ***K ***values [48].

**Figure 5:**
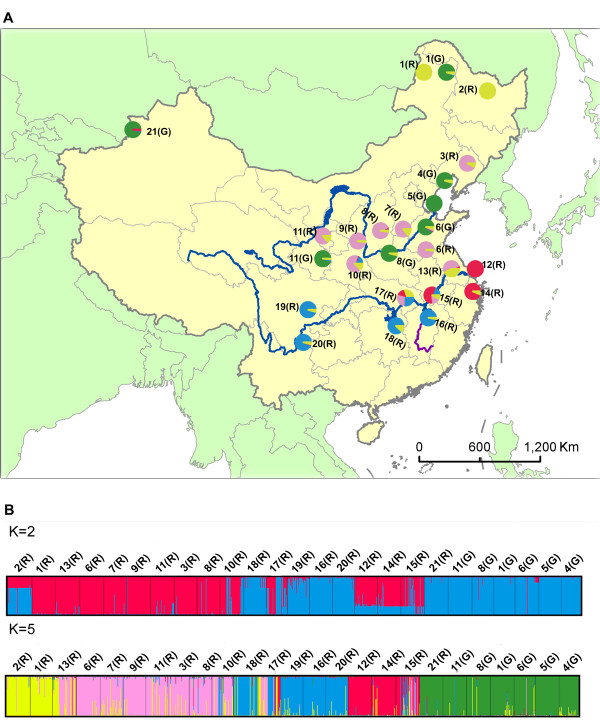
**Clustering analysis by structure for full-loci dataset**. **(A) **Map of *T*. *urticae *population showing the average proportion of the genome inferred by structure to be drawn from each of the five clusters. (**B**) Proportion of the genome of each individual assigned to each of the two clusters and five clusters. Each individual is represented by a vertical bar.

An NJ tree based on DCE genetic distance (Figure 3) for the raw data showed a very high genetic divergence among the 25 *T. urticae *populations, and thus no clear clusters. When the date was corrected by the *INA *method, the NJ tree topology showed subtle differentiation. However, the DCE genetic distance was smaller when calculated with the corrected data than when calculated with the raw data. Moreover, the topology of the tree based on the corrected data (Additional file 4) agrees better with the following Bayesian clustering result than the tree based on the raw data. (The tree branched are coloured according to the Bayesian inferred clusters' colour.) A further STRUCTURE analysis of the full data set (8 loci) revealed the same major patterns that were revealed by the analysis of the five loci with low frequencies of the null alleles. The optimal number of clusters chosen with Evanno's Δ*K *method was five (Figure 4). The pattern of the five clusters corresponds well with the geographical distribution of the populations and the colour form of the mite (Figure 5, Additional file 5). Among the red populations, populations 1 (R) and 2 (R), which are located in the most northern region of China, are grouped in the first cluster and populations 3 (R), 6 (R)-11 (R) and 13 (R) are grouped in the second cluster. All of the populations are north of the Yangtze River except for 13 (R). Populations 16 (R)-20 (R) are grouped in the third cluster. Four out of five populations are located south of the Yangtze River and west of the Gan River. The one exception is 19 (R), which is located north of the Yangtze River. The fourth cluster includes the remaining red mite populations, which are located south of the Yangtze River and east of the Gan River. The populations of green mites were grouped in the fifth cluster. Most populations have a clear allocation to one of the five clusters (i.e., more than 90% of their genome was drawn from one cluster). Figure 5A displays the proportion of each population that contributed to each of the five clusters. Individual assignments are presented in Figure 5B.

AMOVA analysis revealed that the genetic divergence among the five clusters was highly significant (*F*_CT _= 0.31923, *P *< 0.0001; Table 3). This validates the result obtained by Bayesian clustering and means that geographic distance is one of the factors responsible for the genetic structure. Genetic divergence between the red (carmine) and green mites was also highly significant (*F*_CT _= 0.20837, *P *< 0.0001), indicating that the gene flow between the two colour forms in China was limited. The genetic divergence of the mites among the different host plants (*F*_CT _= 0.08941, *P *= 0.00587) was lower than the genetic divergence between the red and green mites, but still significant. Similar results were obtained for the corrected genotype data (Additional file 6).

**Table 3:** AMOVA results comparing genetic variation in 25 *T. urticae *populations collected from 21 localities

Source of variation	d.f	Sum of squares	Variance components	Percentage of variation	Fixation indicies
Among 5 clusters inferred by STRUCTURE	4	1982.124	1.08782 Va	31.92	*F*_CT _= 0.31923 (*P *< 0.0001)

Among population within clusters	20	1310.289	0.75935 Vb	22.28	*F*_SC _= 0.32733 (*P *< 0.0001)

Within populations	2085	3253.593	1.56046 Vc	45.79	*F*_ST _= 0.54207 (*P *< 0.0001)

Among groups with different host plants	4	923.664	0.28920 Va	8.94	*F*_CT _= 0.08941 (P = 0.00587)

Among populations within groups	20	2368.748	1.38490 Vb	42.82	*F*_SC _= 0.47019 (*P *< 0.0001)

Within populations	2085	3253.593	1.56048 Vc	48.24	*F*_ST _= 0.51756 (*P *< 0.0001)

Among groups with different color pattern of mites	1	746.419	0.75113 Va	20.84	*F*_CT _= 0.20837 (*P *< 0.0001)

Among populations within groups	23	2545.994	1.29314 Vb	35.87	*F*_SC _= 0.45316 (*P *< 0.0001)

Within populations	2085	3253.93	1.056048 Vc	43.29	*F*_ST _= 0.567116 (*P *< 0.0001)

## Discussion

Twenty-three of the 25 populations used in this study deviated from HWE, and of these, 19 populations displayed significant heterozygote deficiency. The deficit in heterozygotes may be a universal phenomenon in *T. urticae*. Navajas et al. [12] found heterozygote deficiency within greenhouse populations of *T*. *urticae *populations based on microsatellite markers. Similar observations were made of *T*. *turkestani *Ugarov & Nikolski [14] and *T*. *urticae *along a latitudinal gradient in Europe [15]. However, contrary results were obtained for the corrected genotype data set. After correcting the data set for null alleles, no significant heterozygote deficiency could be detected. This phenomenon suggests that the presence of null alleles is one of the main causes of the deviation from HWE due to heterozygote deficiency. The frequency of null alleles in microsatellite loci seems to be greater in *T. urticae *than in insects [16], probably because of a high rate of mutation in the region flanking the microsatellites [17]. In previous studies of the genetic structure for *T. urtica*e based on microsatellites, the presence of null alleles, inbreeding due to patchy distributions, arrhenotokous mode of reproduction and a Wahlund effect caused by an inaccurate sampling method were considered to be the reasons of departure from HWE [12,15,18,19]. In this study, according to the estimate of the null allele frequencies following the EM algorithm, heterozygote deficiency caused by null allele may be the main factor contributing to the departure from HWE. Additionally, inbreeding, the arrhenotokous mode of reproduction and a Wahlund effect should not be ruled out as contributors to the deviation from HWE. These factors may explain the deviation of HWE for three mite populations [20 (R), 1 (G) and 4 (G)] after genotype was corrected.

The A*R, H*_O_, and *H*_E _values (Table 1) suggests that the genetic diversity within each population of *T. urticae *in China is very low. Considering the demographic and life history traits of *T. urticae *which tend to live in small patches of inbred individuals [12,20], genetic drift probably contributed to the loss of diversity of the mite populations. However, the values of the genetic diversity indexes reported in this study are slightly higher than previously reported values for *T. urticae *[6]. This may be because the latter study used highly inbred laboratory stocks that have low genetic diversity.

*AR *and *H*_E _were significantly greater in the red form mite than in the green form mite (Table 4), suggesting that the genetic diversity of the red form mites was higher. This may be because the carmine spider mite is a native species, while the green spider mite is an invasive species, first reported in Beijing in 1983 [10]. As an invasive pest, the green form is often established in a new area from only a handful of introduced individuals (founders), which carry only a portion of the genetic diversity that was present in the source population. This would explain why the diversity of the green spider mite is lower than that of the carmine spider mite.

**Table 4:** Differences in genetic diversity measure within *T. urticae *populations between the red form mite and the green form mite

	Red form mite	Green form mite	*P *value^a^
Population	18	7	-

*AR*	4.225	3.017	0.011

^R^*H*_E_	0.442	0.297	0.026

^C^*H*_E_	0.501	0.343	0.015

^R^*F*_IS_	0.284	0.151	0.129

^C^*F*_IS_	-0.051	-0.022	0.265

^R^*F*_ST_	0.418	0.468	0.140

*F*_ST _^{*ENA*} ^	0.383	0.434	0.106

Population Number of populations include in each form; allelic richness for standardized samples of 68 gene copies (*AR*); expected heterozygosity calculated by the raw data and corrected data (^R^*H*_E_/^C^*H*_E_); fixation index calculated by the raw data and corrected data (^R^*F*_IS_/^C^*F*_IS_); coefficients of genetic differentiations among populations calculated by the raw data (^R^*F*_ST_). coefficients of genetic differentiations among populations calculated using the *ENA *method. (*F*_ST _^{*ENA*}^)

^a ^Probalities associated with permutation tests for difference in *AR*, ^R^*H*_E_, ^C^*H*_E_, ^R^*F*_IS_, ^C^*F*_IS_, ^R^*F*_ST_, *F*_ST _^{*ENA*} ^, and with a *t *- test for paired comparison.

The pairwise *F*_ST _values, Cavalli-Sforza & Edwards' chord distances, Bayesian clustering and AMOVA structure suggest that *T. urticae *has a high level of genetic structuring. Although there is a strong subdivision of genetic structure in Chinese populations of *T. urticae*, low gene flow still occurred among geographical populations (Figure 5B). This may be due to the long distance dispersal facilitated by multiple dispersal mechanisms of *T*. *urticae*. Five clades likely exist in China. The red form mite populations were clustered into four clades which agree well with their geographical distribution. The limitation of gene flow between populations may play a role in the high genetic differentiation. Our results suggest that the Yangtze River doesn't act as a substantial barrier to gene flow in the red form mite populations, which was contrary to be the case of the rice stem borer, *Chilo suppressalis *[21]. However some studies also showed little or no evidence that the Yangtze River limits gene flow [22,23]. The different characteristics of different dispersal mechanisms of organisms may be responsible for this difference. For *T. urticae*, geographic distance may be the prevailing factor for the limited gene flow as evidenced by the IBD analysis. IBD analysis revealed that geographic distance has a strong effect on the population structure of the red form mite (R^2 ^= 0.1129, *P *= 0.0050) and no effect on the green form mite (R^2 ^= 0.001, *P *= 0.42800). The limitation of gene flow associated with geographical distance is in agreement with the results of previous studies of spider mites [14,24,25].

The declining genetic diversity of the red form mite with increasing latitude may be because the northern populations have fewer generations per year. In southern China, which has a hot-humid climate, the spider mite has more than 20 generations per year whereas in northern China, which has a cold-arid climate, the spider mite has 12-15 generations per year. This would result in a higher mutation rate in the southern populations. Mutation is one of the key sources of genetic diversity. So the southern populations would be expected in turn to have a higher genetic diversity than the northern populations. However, only seven populations of green mites were considered in this study, and all of the seven populations were located in the north of China. Therefore, scarce populations of green mites scattered over a much lower latitudinal gradient than the populations of red mites might lead to the statistical bias.

Even though *T. urticae *and *T. cinnabarinus *have significantly different body colours and are reproductively isolated in China, our genetic data don't support the separation of the two forms of spider mite into two species. The level of genetic differentiation between the two colour forms is similar to that examined between geographically separated populations. Bayesian clustering cannot separate the red form mite from the green form mite when K = 2. AMOVA analysis also revealed that there is more variations among the 5 groups inferred by STRUCTURE software than between the two colour forms of spider mite. An attempt to distinguish the two forms by phylogenetic analysis of their endosymbiont *Wolbachia *was unsuccessful, possibly because of a tendency of *Wolbachia *to co-evolve with their hosts [26]. Although Li et al. reported that they could differentiate the red and green mites using three microsatellite markers, three red form mite populations were still mixed with green form mite populations in their results [6]. Based on part of mitochondrial (cytochrome c oxidase subunit 1) and nuclear (internal transcribed spacer 1 and 2 of ribosomal RNA gene) sequences, the relationship between the two species also cannot be well resolved [27]. The completely incompatible between the two forms of mite may be because the two forms were isolated from each other for a long time before the green mite form was introduced to China. A long period of isolation leads to complete reproductive incompatibility. Although *T. urticae *and *T. cinnabarinus *are reproductively incompatible, there may be no intrinsic difference in their DNA compositions. The morphological difference between the two forms of mite may be due to epigenetics. In this case, different gene expressions between the two forms of spider mite caused by DNA methylation, histone deacylation and other epigenetic factors may lead to the different phenotypes. Thus, the two forms of *T. urticae *may be good candidates for epigenetic studies.

## Conclusions

Spider mites possess low levels of genetic diversity, limit gene flow between populations and significant IBD (isolation by distance) effect. These factors in turn contribute to the strong subdivision of genetic structure. Due to the founder effect, the genetic diversity of the invasive green form of spider mite is lower than that of the red form. Fewer generations per year at higher latitude lead to the declining genetic diversity of the red form with increasing latitude. In addition, the population genetic structures of the two forms of mites do not support their separation into two species. The morphological difference between the two forms of mites may be the result of epigenetic effects.

## Methods

### Mite sampling and DNA extraction

**Figure 6:**
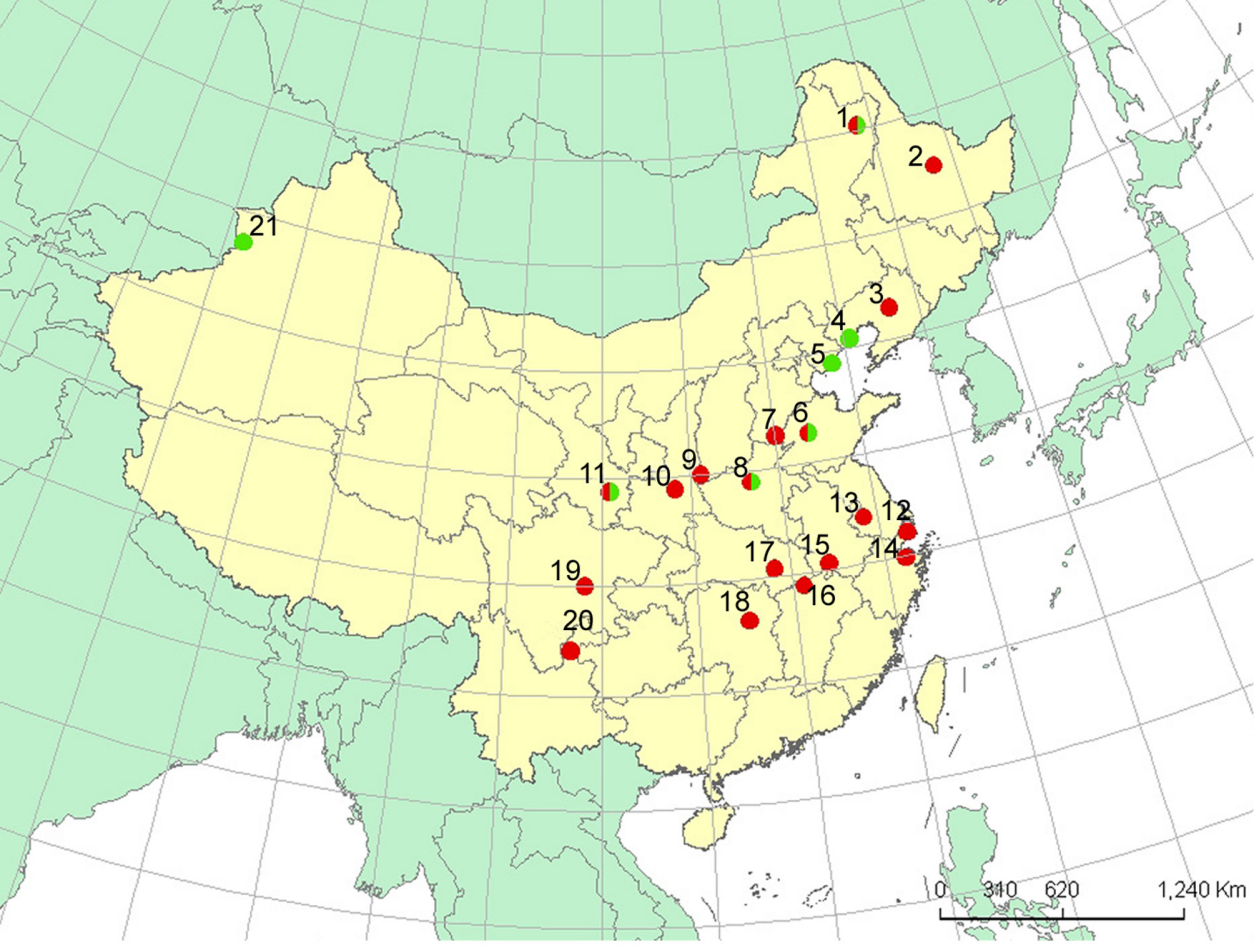
**Sample locations of 25 Tetranychus urticae populations used in this study**. Red and green dots indicate red and green form mite populations, respectively, and dual colours dots represent the locality where the two forms of mite populations were sampled. 1. Neimenggujiagedanai (JGDN) 124°04'E,50°24'N; 2. Heilongjiangyichun(HLJYC) 128°56'E, 47°42'N; 3. Liaoningshenyang (LNSY) 123°25'E, 41°48'N; 4. Liaoningxingcheng (LNXC) 120°41'E, 40°37'N; 5. Hebeichangli (HBCL) 119°09'E, 39°42'N; 6. Shandongjinan (SDJN)117°32'E, 36°43'N; 7. Hebeiqiuxian (HBQX) 115°1'E, 36°49'N; 8. Henanzhengzhou (HNZZ) 113°4'E, 34°46'N; 9. Shanxiyuncheng (SXYC) 110°59'E, 35°02'N; 10. Shanxiyanliang (SXYL) 109°12'E, 34°4'N; 11. Gansutianshui (GSTS) 105°42'E, 34°37'N; 12. Shanghainanhui (SHNH) 121°45'E, 31°03'N; 13. Jiangsuzhenjiang (JSZJ) 119°27'E, 32°11'N; 14. Zhejiangcixi (ZJCX) 121°15'E, 30°11'N; 15. Anhuianqing (AHAQ) 117°02'E, 30°31'N; 16. Jiiangxijiujiang (JXJJ) 115°58'E, 29°43'N; 17. Hubeiwuhan (HBWH) 114°17'E, 30°35'N; 18. Hunanchangsha (HNCS) 112°59'E, 28°12'N; 19. Sichanmeishan (SCMS) 104°08'E, 30°00'N; 20. Yunnanyulong (YNYL) 103°42'E, 27°05'N; 21. Xinjiangzining (XJZN) 81°2'E, 43°55'N.

During the summer of 2008, we collected a total of 1055 adult females of the two-spotted spider mite (the green form from 7 regions, and the red form from 18 regions) at 25 sites in China. Figure 6 provides information about the sampling localities. The host plants and the number of analysed mites are summarised in Table 1. At each locality, every population was sampled by randomly collecting 40-50 adults from 20 plants in a 5- × 5-m square. Each locality was > 300 km away from the others. They were brought to the laboratory for identification based on aedeagus morphology observed with a V8 microscope (Carl Zeiss, Jena, Germany). Only *T. urticae *mites were subjected to further analyses. Total DNA was extracted from adult female mites according to the method of Gomi et al. [28].

### Microsatellite loci isolation and characterization

Two of the eight loci used in this study (TECI 04 and TECI 08) were isolated by us from a genomic DNA library using a suppression - PCR procedure described by Lian et al. [29]. Briefly, DNA extracted from a pool of mites was digested with a blunt - end restriction enzyme, *EcoRV*, and the restricted fragments were then ligated with a specific blunt adaptor (consisting of a 48-mer: 5'GTAATACGATTCACTATAGGGCACGCGTGGTCGACGGCCCGGGCTGGT3' and 8-mer with the 3'-end capped by an amino residue: 5 ACCAGCCC-NH_2_3') by use of a DNA ligation kit (Takara Shuzo, Japan).

Fragments were amplified from an *EcoRV *DNA library using compound SSR primer (AC)_6_(AG)_5 _or (AG)_6_(AC)_5 _and an adaptor primer AP2 (5'CTATAGGGCACGCGTGGT3'). The amplified fragments were integrated into the plasmids using a pT7 Blue Perfectly Blunt Cloning Kit (Novagen) and the plasmids were transferred into *Escherichia coli *according to the manufacturer's instructions. The inserted fragment lengths were checked by 1.5% agarose gel electrophoresis. Amplified fragments between 300 and 800 base pairs were selectively sequenced directly using a Thermo Sequence Pre - mixed Cycle Sequencing Kit (Amersham Biosciences, USA) with a Texas Red-labeled T7 primer (Sigma-Aldrich, Japan) in an SQ-5500E sequencer (Hitachi). For each fragment flanking (AC)_6_(AG)n or (AG)_6_(AC)n compound SSR sequences at one end, a specific primer (IP1) was designed from the sequence using Primer 5.0 (http://www.premierbiosoft.com). The primer pairs of IP1 and compound SSR primer were used as a compound SSR marker.

To examine the effectiveness of primer pairs designed as compound SSR markers, 40*T. urticae *adult females sampled from 21 provinces of China were used for the template DNA extraction according to protocols described by Gomi et al. [28]. PCR amplification was conducted with a Gene Amp PCR System 9700 (Applied Biosystems). Five microliters of the reaction mixture contained 0.5 μL template DNA, 0.2 mmol^-1 ^of each dNTP, 1 × PCR buffer (Mg^2+ ^free, Applied Biosystems, USA), 2.5 mmol L^-1 ^Mg^2+^, 0.125 U of Ampli *Taq *Gold (Applied Biosystems), and 0.5 μmol L^-1 ^of each IP1 and a Texas Red-labeled compound SSR primer ((AC)_7_(AG)_3 _or (AG)_7_(AC)_3_). Reactions were preceded by a 9-min denaturation step at 94°C and were cycled 40 times with 30s at 94°C, 30s at 55°C, and 1 min at 72°C, followed by a final 5 min extension step at 72°C. The reaction products were electrophoresed on 6% Long Ranger sequencing gel (FMC BioProducts, ME, USA) using an SQ - 5500E sequencer. Electrophoretic patterns were analysed by Fraglys v.3 (Hitachi).

### Genotyping

We scored eight microsatellite loci: TU1, TU11, TU35b [12], TUCA12, TUCT17, TUCA72 [13], and TECI04, TECI08 (newly found in our study). Tests for pair-wise compatibility of the primers found there's no interaction between any pairs of the eight loci. Therefore, all loci were amplified jointly in a final volume of 5 μl, using the multiplex polymerase chain reaction *Taq *from QIAGEN following the manufacturer's recommendations. Amplifications were conducted on Gene Amp PCR System 9700 (Applied Biosystems), and genotyping was performed on an ABI 3100 sequencer. Genotypes were then checked by eye with GeneMapper 3.0 software (Applied Biosystems).

### Statistical analysis

Micro-Checker software (Ver. 2.2.3) [30] was first used to examine null alleles with the Oosterhout algorithm. Linkage disequilibrium between all pairs of locus were tested with FSTAT 2.9.3.2 [31]. To assess the genetic diversity within each population, we used FSTAT to calculate (1) the number of alleles (*A*), (2) the allelic richness [32] based on a minimum population size of 34 diploid individuals (68 gene copies; *AR*), and (3) the fixation index (*F*_IS_) at each locus and over all loci in each population. The deviation of *F*_IS _from zero was tested in each population by 1000 permutation tests with a sequential Bonferroni correction. The observed heterozygosity (*H*_O_) and expected heterozygosity *(H*_E_) were calculated at each population using GenALEX 6 software [33]. Deviation from Hardy-Weinberg equilibrium (HWE) [34] at each locus for each population was tested with GENEPOP v.3.4 [35]. *P *values were corrected for multiple comparisons by applying a sequential Bonferroni correction [36]. To minimize the bias of the genetic diversity statistics induced by null alleles, genotypes were corrected according to the null frequencies estimated by the EM algorithm of Dempster et al. (1977) [37] implemented in the program FREENA (http://www.montpellier.inra.fr/URLB/) [38]. The estimated false homozygous genotypes XX caused by null alleles were systematically changed to X999 (*INA *method) [38-40]. The corrected data set was used to rectify *H*_O_, *H*_E_, *F*_IS _statistics and HWE test using the above methods.

In order to understand whether genetic variation within populations is correlated with geographical gradients, Pearson correlations between the statistics of variation (*AR *and *H*_E_) and geographic co-ordinates (latitude and longitude) for each population were analysed. Stepwise regression analysis of *AR *and *H*_E _in relation to the two independent variables (latitude and longitude) were further assessed separately. Both analyses were conducted using SPSS 13.0 for Windows [41].

Pairwise *F*_ST _values for each population comparison were calculated with FSTAT. The *ENA *method was also used to obtain the unbiased pairwise *F*_ST _values (*F*_ST _^{*ENA*}^) using the FREENA program [38]. To detect isolation-by-distance effects, we compared *F*_ST_/(1-*F*_ST_) matrix and *F*_ST _^{*ENA*}^/(1-*F*_ST_^{*ENA*}^) matrix with a geographic distance matrix (ln Km) using the Mantel test, with significance tests performed over 1000 permutations [42]. The test was implemented in GENEPOP [35].

Phylogenetic relationships among populations were estimated by constructing a neighbour-joining (NJ) tree [43] based on DCE distance [44] using the PHYLIP 3.6c package [45]. One thousand distance matrices from resampled data sets bootstrapped over allele frequencies were created using the SEQBOOT subroutine in PHYLIP3.6c. Then the GENDIST subroutine was used to calculate the correlated genetic distances. The distances matrices were used to construct NJ trees using the NJ tree NEIGHBOUR subroutine in PHYLIP 3.6c. The input order was randomised to ensure the final tree topology was not dependent on the sample entry order. The CONSENSE subroutine within PHYLIP produced a consensus NJ tree that provided estimates of robustness at each node based on the bootstrapping of the gene frequencies. Consequently, we constructed a visual tree with TREEVIEW v.1.6.6 [46]. Because the presence of null alleles may confound the calculation of genetic distance, the corrected data using the *INA *method implemented in FREENA was also used to constructing a NJ tree using the above methods.

The Bayesian clustering method was also used to elucidate the genetic structure among populations using STRUCTURE v. 2.2 [47]. The model applied in the analysis assumes the existence of *K *clusters. We took advantage of an admixture ancestry model under the correlated allele frequency model. The Markov chain Monte Carlo simulation was run 20 times for each value of *K *(1-11) for 10^6 ^iterations after a burn-in period of 10^5^. All other parameters were set at their default values. We used the Δ *K *method of Evanno et al. [48] to choose the most likely value of *K*. The proportional membership of each cluster was estimated for each individual and each population. Owing to the difficulty of correcting multilocus genotypes precisely, three loci, TUCA12, TUCT17, TUCA72 abounded with null alleles over the 18 population were deleted. The genotype data with the remaining five loci were also used for Bayesian clustering to exclude the effect of null alleles. After the three loci were deleted, 79 of 125 locus-population combinations were found to have no null alleles. The average frequency of null alleles for the remaining five loci was estimated at 0.083 by MICRO-CHECKER software.

Population genetic variance was further analysed by an Analysis of Molecular Variance (AMOVA) [49] performed by the method of Excoffier et al. [49] using ARLEQUIN v. 3.11 [50]. Both the raw genotype data set and the genotype data set which corrected by using the *INA *method were analysed. Genetic variance was partitioned into three levels: (1) among different groups defined on the basis of phylogenetic clusters, host plants, and mite colour patterns, (2) among populations within groups, and (3) within populations. Significance of fixation indices was tested using a nonparametric permutation approach with 1000 permutations [49], as performed by ARLEQUIN.

## Authors' contributions

XYH JTS participated in the design of the study. JTS CL performed the experiments. CL JTS participated in genetic data analysis. JTS MN XYH drafted the manuscript. All authors read and approved the final manuscript.

## Supplementary Material

Additional file 1 **Pearson correlations between **^**C**^***H***_**E **_**and geographic latitude**. Expected heterozygosity calculated by the corrected data (^C^*H*_E_); R = -0.469, *P *< 0.05. Click here for file

Additional file 2 **Pairwise *F*_ST _^{*ENA*} ^values between all populations**. Click here for file

Additional file 3 **Scatter plots of *F*_ST _^{*ENA*} ^vs. geographical distance for pairwise population comparisons**. Click here for file

Additional file 4 **Consensus neighbour-joining tree based on DCE distances calculated on corrected data using the *INA *method**. Click here for file

Additional file 5 **Clustering analysis by structure for five-loci dataset**. Click here for file

Additional file 6 **AMOVA results of the corrected data set using the *INA *method**. Click here for file
