# Supplementary figures and images for: Microsatellites reveal a strong subdivision of genetic structure in Chinese populations of the mite Tetranychus urticae Koch (Acari: Tetranychidae)
Source: BMC Genet. 2012 Feb 21;13:8. doi: 10.1186/1471-2156-13-8 (PMC3309930; doi:10.1186/1471-2156-13-8)

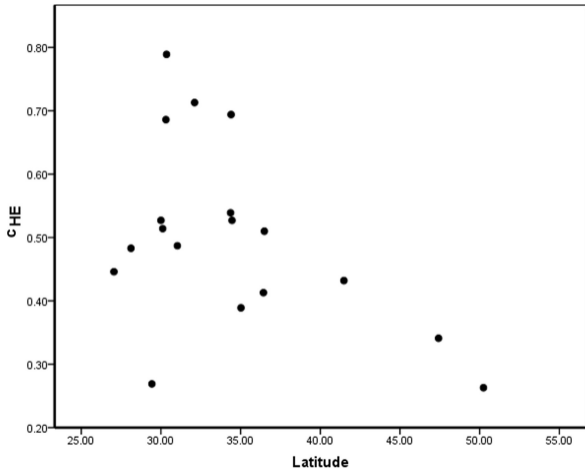

Supplement: Additional file 1 — Pearson correlations between CHE and geographic latitude. Expected heterozygosity calculated by the corrected data (CHE); R = -0.469, P < 0.05. [file 1471-2156-13-8-S1.PDF]

**A**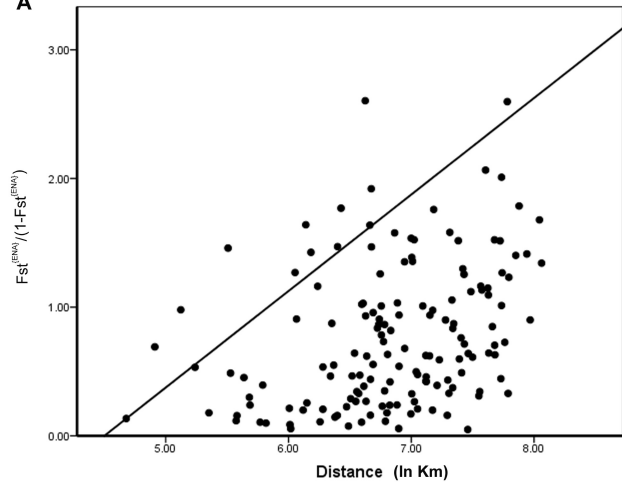**B**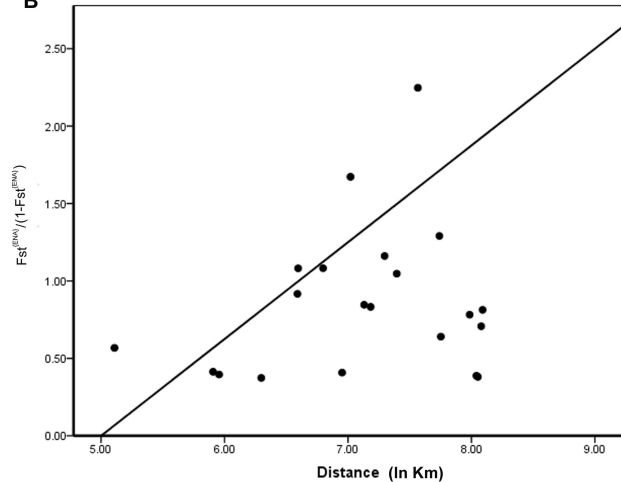

Supplement: Additional file 3 — Scatter plots of FST {ENA} vs. geographical distance for pairwise population comparisons. [file 1471-2156-13-8-S3.PDF]

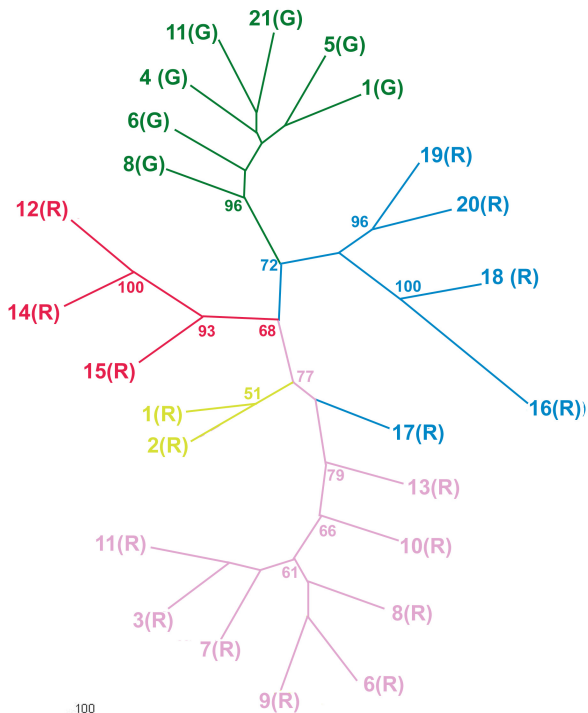

Supplement: Additional file 4 — Consensus neighbour-joining tree based on DCE distances calculated on corrected data using the INA method. [file 1471-2156-13-8-S4.PDF]

2(R) 1(R) 13(R) 6(R) 7(R) 9(R) 11(R) 3(R) 8(R) 10(R) 18(R) 17(R) 19(R) 16(R) 20(R) 12(R) 14(R) 15(R) 21(R) 11(G) 8(G) 1(G) 6(G) 5(G) 4(G)

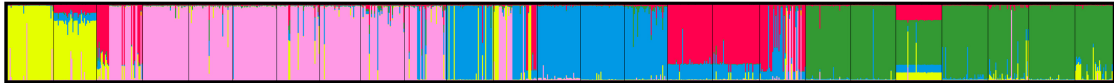

Supplement: Additional file 5 — Clustering analysis by structure for five-loci dataset. [file 1471-2156-13-8-S5.PDF]
